# The regulation of β-catenin activity and function in cancer: therapeutic opportunities

**DOI:** 10.18632/oncotarget.15687

**Published:** 2017-02-25

**Authors:** Shuang Shang, Fang Hua, Zhuo-Wei Hu

**Affiliations:** ^1^ Immunology and Cancer Pharmacology Group, State Key Laboratory of Bioactive Substance and Function of Natural Medicines, Institute of Materia Medica; Chinese Academy of Medical Sciences & Peking Union Medical College, Beijing, P.R. China

**Keywords:** Wnt signaling, protein stability, subcellular localization, transcriptional regulation, cancer therapy

## Abstract

Wnt/β-catenin signaling is an evolutionarily conserved and versatile pathway that is known to be involved in embryonic development, tissue homeostasis and a wide variety of human diseases. Aberrant activation of this pathway gives rise to the accumulation of β-catenin in the nucleus and promotes the transcription of many oncogenes such as *c-Myc* and *CyclinD-1*. As a result, it contributes to carcinogenesis and tumor progression of several cancers, including colon cancer, hepatocellular carcinoma, pancreatic cancer, lung cancer and ovarian cancer. β-Catenin is a pivotal component of the Wnt signaling pathway and it is tightly regulated at three hierarchical levels: protein stability, subcellular localization and transcriptional activity. Uncovering the regulatory mechanisms of β-catenin will provide new insights into the pathogenesis of cancer and other diseases, as well as new therapeutic strategies against these diseases. In this review we dissect the concrete regulatory mechanisms of β-catenin from three aspects mentioned above. Then we focus on the role of β-catenin in cancer initiation, progression, dormancy, immunity and cancer stem cell maintenance. At last, we summarize the recent progress in the development of agents for the pharmacological modulation of β-catenin activity in cancer therapy.

## INTRODUCTION

β-Catenin is a multifunctional protein with a central role in physiological homeostasis. Its aberrant high expression leads to various diseases including cancer. β-Catenin is the mammalian homologue of the drosophila armadillo gene. It acts both as a transcriptional co-regulator and an adaptor protein for intracellular adhesion. Wnt is the chief regulator of β-catenin, which is a family of 19 glycoproteins to regulate both the β-catenin-dependent (canonical Wnt) and -independent (non-canonical Wnt) signaling pathways [1]. In canonical Wnt pathway, Dsh, β-catenin, Glycogen Synthase Kinase 3 beta (GSK3β), adenomatous polyposis coli (APC), AXIN, and T-cell factor (TCF)/lymphoid enhancement factor (LEF) have been identified as signal transducers of the canonical Wnt pathway, in which β-catenin is a core molecule [2–5]. In the absence of Wnt ligands, β-catenin is kept at a low level through the ubiquitin proteasome system (UPS). β-Catenin is recruited into a destruction complex that contains APC and AXIN, which facilitates the phosphorylation of β-catenin by casein kinase 1 (CK1) and then GSK3β. This leads to the ubiquitylation and proteasomal degradation of β-catenin. Upon Wnt activation or genetic mutations of Wnt components, β-catenin accumulates in the cytoplasm and then translocates into the nucleus. Consequently, it binds to LEF-1/TCF4 and some other co-regulators to promote the transcription of target genes such as *Jun*, *c-Myc* and *CyclinD-1* in a tissue specific manner, most of which encode oncoproteins. In more than half of all cancer cases, such as colorectal carcinoma, breast cancer, liver carcinoma, melanoma and leukemia, β-catenin accumulates within the nucleus or cytoplasm [6–10]. It helps to maintain the stemness of normal intestinal cells, furthermore, high-level cytoplasm expression and nuclear localization of β-catenin always induces tumorigenic traits and promotes cancer cell proliferation and survival [11]. Besides, β-catenin promotes the progression of tumors via suppressing the T-cell responses [12]. The activity of β-catenin is controlled by a large number of binding partners that affect its stability, cellular localization and transcriptional activity. Understanding these regulatory mechanisms is of great importance for the identification of potential therapeutic targets to fight against Wnt/β-catenin-related cancers. This review focuses on the regulation of β-catenin activity in the canonical Wnt/β-catenin signaling pathway. We will provide an overview of the molecular mechanisms involved in the regulation of β-catenin signaling and the compelling therapeutic strategies aimed at targeting the Wnt/β-catenin pathway, which show promising anti-tumor activity.

## REGULATION OF β-CATENIN STABILITY

The stabilization of β-catenin is critical in tumorigenesis, which is usually induced by aberrant Wnt activation and somatic gene mutations of β-catenin or destruction complex components [13, 14]. With inactivation of the β-catenin-destruction complex, β-catenin accumulates in the cytoplasm, and eventually translocates into the nucleus, where it binds with the TCF/LEF family members and induces the transcription of target genes. Indeed, nuclear accumulation of β-catenin can be observed in 80% of colorectal carcinoma [15]. The UPS-dependent degradation is considered to be the major mechanism for controlling the stability of β-catenin. However, it has been recently reported that β-catenin forms a complex with LC3 for an autophagy-dependent degradation [16].

### β-Catenin and the ubiquitin-proteasome pathway

#### The canonical destruction complex of β-catenin

In the absence of Wnt, β-catenin is delivered to proteasome for degradation by the so-called cytoplasmic “destruction complex”, which is composed of APC, Axin (Axin-1, Axin-2), GSK3β, and casein kinase 1α (CK1α) [17]. APC recruits β-catenin to the destruction complex [18]. After being phosphorylated by GSK3β or CK1, the phosphorylated APC recruits unphosphorylated β-catenin to the destruction complex. After being dephosphorylated by protein phosphatase 2A (PP2A), APC “hands” β-catenin to Axin to undergo phosphorylation and ubiquitination [19]. Axin functions as the core molecule of the complex that physically interacts simultaneously with β-catenin, APC and GSK3β [20]. GSK3β phosphorylates β-catenin at Ser33, Ser37 and Thr41 after the priming phosphorylation of β-catenin at Ser45 by CK1α, and this exposes the phosphorylated β-catenin to E3 ligases [21, 22]. β-Catenin or APC are considered as the “gatekeepers” of Wnt signaling, mutations of which usually occur early during tumorigenesis [23, 24]. Frizzled is a family of G protein-coupled receptor proteins. Frizzled and low-density lipoprotein receptor-related proteins 5/6 (LRP5/6) act as Wnt receptors. The formation of the FZ and LRP5/6 heterodimer is an indispensable event in the activation of Wnt signaling [25, 26]. In the presence of Wnt, phosphorylated LRP5/6 provides a binding site for Axin and GSK3β through the PPPsPxS motifs, which disassembles the destruction complex and inhibits the degradation of β-catenin [27, 28].

#### Phosphorylation and degradation of β-catenin

**Figure 1:**
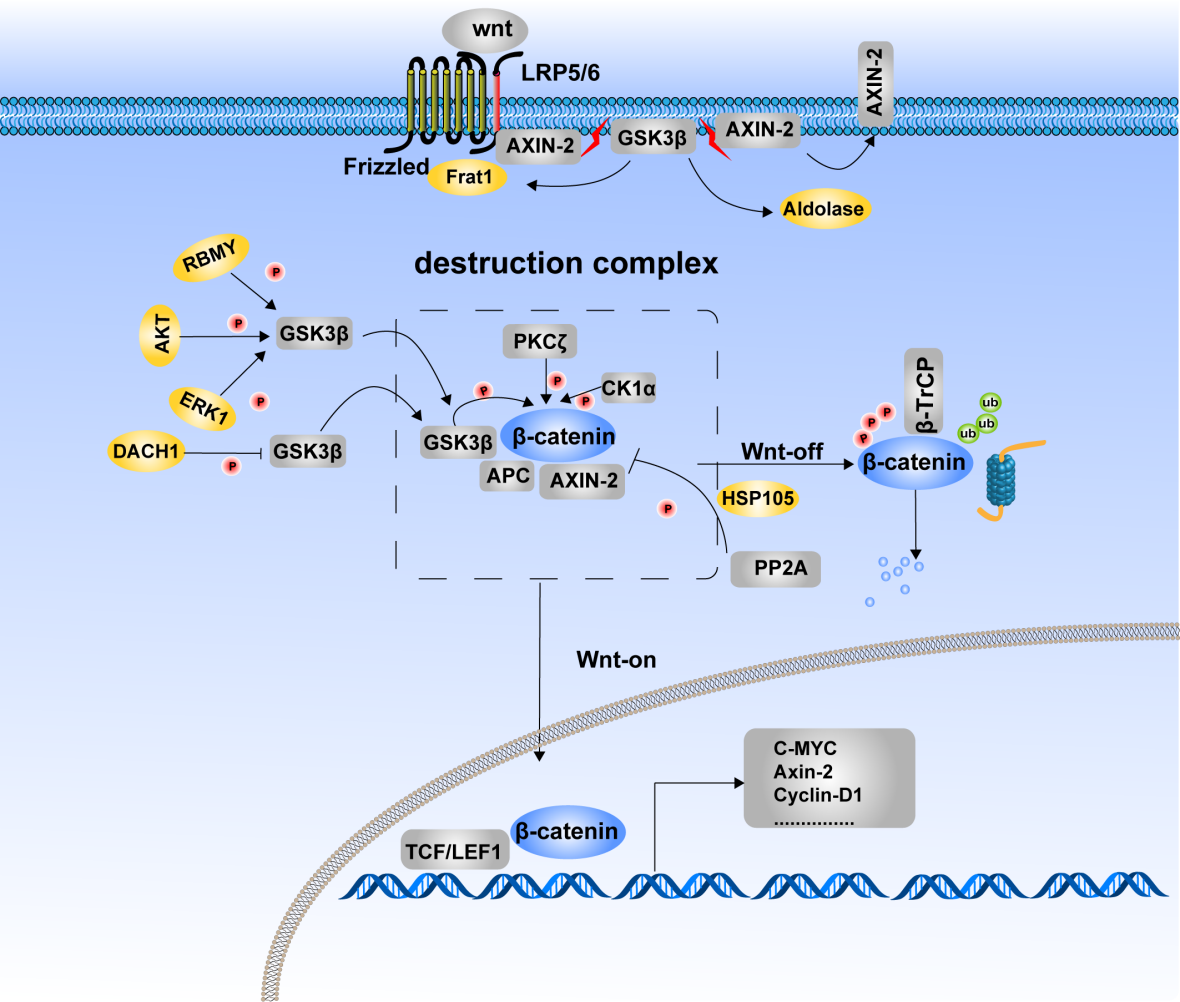
Phosphorylation of β-catenin and its degradation The phosphorylated status of β-catenin is determined for its stability. Destruction complex formation is the requested step for β-catenin phosphorylation. In the Wnt-off state, β-catenin was recruited to the destruction complex and induced phosphorylation and subsequent degradation. In the Wnt-on state, β-catenin translocates into the nucleus due to the disassembly of the destruction complex. LRP5/6, FRAT1 and aldolase inhibit β-catenin degradation by suppressing assembly of the destruction complex, which provides a platform for β-catenin phosphorylation. Kinases and phosphatases jointly take part in the balance control of β-catenin between phosphorylation and dephosphorylation. GSK3β, CK1α and PKCζ are protein kinases that catalyze β-catenin phosphorylation and its subsequent degradation. PP2A is the phosphatase that induces dephosphorylation of β-catenin and inhibits its degradation. RBMY, AKT, ERK1, and DACT1 indirectly regulate the phosphorylation and degradation of β-catenin by modulating the Ser9 phosphorylation of GSK3β to inactivate its kinase activity.

A central question in Wnt-signaling transduction is the regulation of β-catenin phosphorylation, in which kinases and phosphatases jointly take part. Thus, a balance between phosphorylation and dephosphorylation must be reached. PP2A is a heterotrimeric complex, in which PR55α (a regulatory subunit) suppresses the function of GSK3β by dephosphorylating β-catenin [29]. Phosphorylation of β-catenin is fine-tuned by direct phosphorylating β-catenin itself or by modulating its kinases or phosphatases (Figure 1).

Destruction complex formation is a required step for β-catenin phosphorylation. LRP6, frequently rearranged in advanced T-cell 1 (FRAT1) and aldolase are proteins involved in the regulation of destruction complex formation. As a receptor of Wnt, LRP6 acts as an inhibitor of GSK3β in response to Wnt stimulation [30]. Upon activation by Wnt, LRP6 is phosphorylated, resulting in the recruitment of Axin1 to LRP6. As a result, phosphorylation of β-catenin by GSK3β is inhibited and β-catenin is stabilized [31]. FRAT1, a component of the Wnt/β-catenin signaling pathway, is positively correlated with tumor progression. FRAT1 is reported to prevent GSK3β from binding to the Axin1-APC-CK1 complex by competing with Axin1 for a common or closely overlapping binding site on GSK3β [32]. Aldolase has a similar function as FRAT1. Aldolase family members were recently reported to be prognostic markers of colorectal cancer progression. Through binding with GSK3β, aldolase disrupts the GSK3β–Axin interaction, leading to membrane translocation of Axin and activation of Wnt signaling [33].

Protein kinase Cζ (PKCζ) is another protein kinase that phosphorylates Wnt/β-catenin signaling through direct phosphorylation of β-catenin at Ser45 in a CK1α-independent manner and promoting β-catenin degradation. This effect represses intestinal stem cell function and tumorigenesis [34]. Depletion of heat shock protein 105 (HSP105) a required component of the Wnt/β-catenin pathway, results in β-catenin accumulation and target gene transcription following Wnt stimulation. Mechanistically, HSP105 promotes the integration of PP2A into the β-catenin degradation complex, thus antagonizes the phosphorylation and degradation of β-catenin [35].

Some proteins indirectly regulate β-catenin phosphorylation and degradation through modulating GSK3β kinase activity. Dachshund inactivates Wnt signaling and inhibits hepatocellular carcinoma (HCC) growth through decreasing GSK3β Ser9 phosphorylation [36]. The later effect maintains GSK3β kinase activity and promotes β-catenin degradation. RNA-binding motif on Y chromosome (RBMY) is overexpressed in male HCC tissues. RBMY induces Ser9 phosphorylation-induced inactivation of GSK3β, thereby impeding the GSK3β-mediated degradation of β-catenin and subsequently increasing stemness of HCC [37]. Besides RBMY, HBx-activated AKT and ERKs also phosphorylate and inactivate GSK3β, leading to stabilization of β-catenin and *CyclinD1* gene transcription, and promoting the development of HCC [38].

#### Ubiquitination and degradation of β-catenin

**Figure 2:**
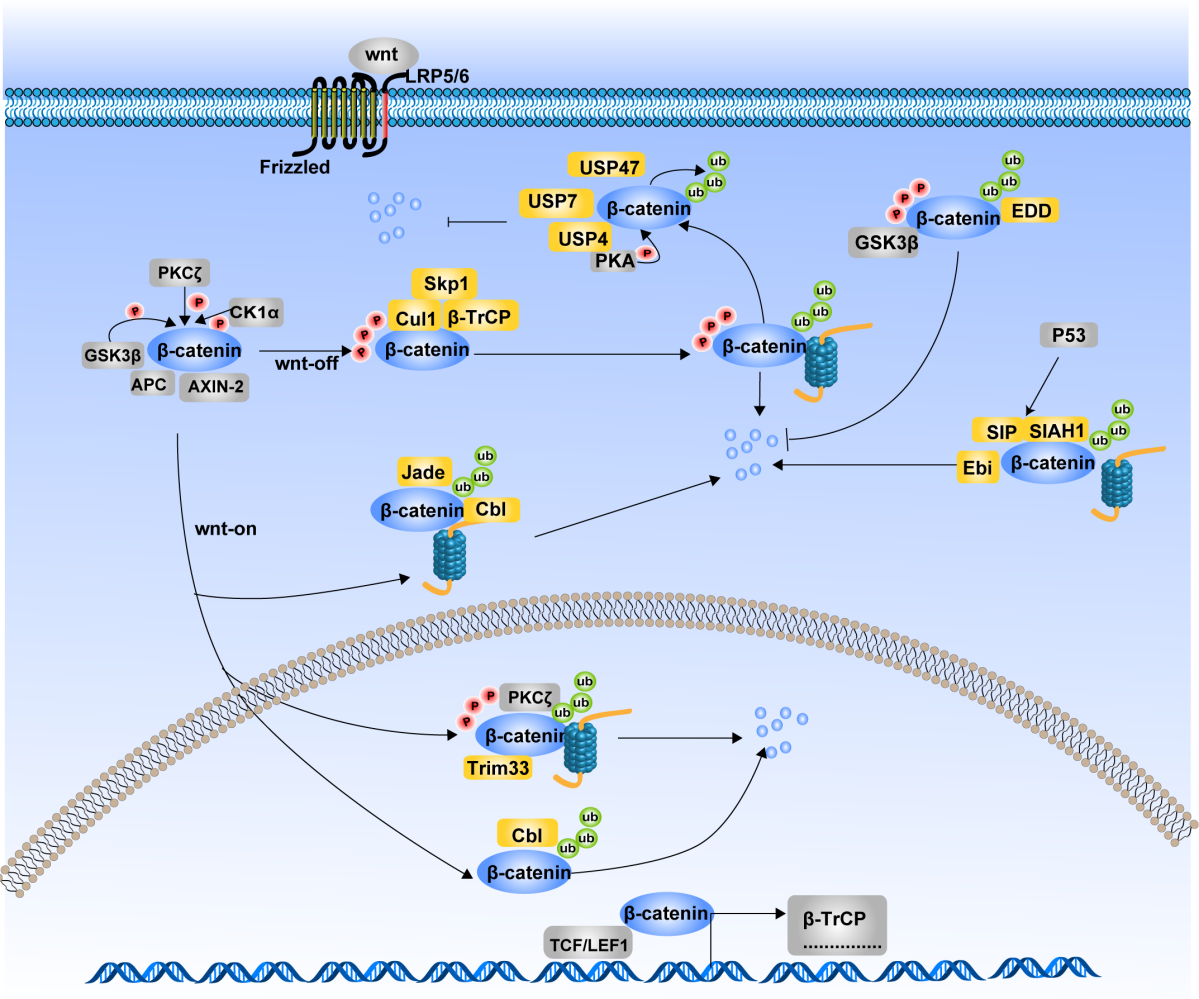
Ubiquitination of β-catenin and its degradation The degradation of β-catenin mainly undergoes through Ubiquitin Proteosome System (UPS). Different ubiquitin E3 ligases in the cytoplasm and nucleus regulate the ubiquitination of β-catenin. Ubiquitination of β-catenin can be occurred either in the Wnt-off state or in the Wnt-on state. Together with Cul1 and Skp1, β-TrCP mediates the ubiquitination of β-catenin in the Wnt-off state. While Jade1, c-Cbl and Trim33 induce ubiquitination of β-catenin in the Wnt-on state. Additionally, SIAH1 along with SIP and Ebi, ubiquitinate β-catenin in response to p53 activition. Phosphrylation state is not always a necessary condition for β-catenin ubiquitination. β-TrCP and Trim33-induced ubiquitination of β-catenin is phosphorylation dependent, while ubiquitination of β-catenin by Jade-1, c-Cbl and SIAH1 are phosphorylation independent. In contrast to afore mentioned E3 ligases, EDD ubiquitinates β-catenin with an inhibitory effects on its degradation. Several deubiquitinases such as USP4 and USP7 can deubiquitinate β-catenin and interrupt its degradation process.

The ubiquitination of β-catenin is a complex process, which is either phosphorylation dependent or independent. Also, this post-translational modification happens either in the cytoplasm or in the nucleus (Figure 2). Actually, phosphorylated β-catenin is recognized and ubiquitinated by specific E3 ubiquitin ligases, and undergoes degradation in the proteasome. The degradation of β-catenin could happen both in the cytoplasm and in the nucleus according to Wnt status. In the “Wnt-off” state, β-catenin is mainly degraded in the cytoplasm, whereas in the “Wnt-on” state, β-catenin is mainly degraded in the nucleus via the UPS. The corresponding E3 ubiquitin ligases are different at different subcellular locations. In the cytoplasm, β-TrCP, Jade1, casitas B-lineage lymphoma (c-Cbl) and the human homolog of drosophila seven in absentia (SIAH1) are E3 ligases that mediate β-catenin ubiquitination. Of these E3 ligases, only β-TrCP and Jade1 are Wnt responsive. β-TrCP interacts with the Cul1/Skp1 complex as well as β-catenin, thus to ubiquitinate β-catenin. In turn, Wnt/β-catenin signaling induces the expression and activity of β-TrCP ubiquitin ligase receptor and this negative feedback loop controls the β-catenin/TCF4 pathway [39]. Moreover, induction of the β-TrCP–GSK3β interaction is required for late-phase stabilization of β-catenin in canonical Wnt signaling. Jade1 is a candidate renal tumor suppressor that binds to the N-terminus of β-catenin in a Wnt-responsive fashion. Either phosphorylated or non-phosphorylated β-catenin can be ubiquitinated by Jade1. β-Catenin Ser33 phosphorylation induced by GSK3β enhances its binding with Jade1 in the Wnt-off state [40]. Thus different characteristics of β-TrCP and Jade1 ensure optimal cytoplasmic β-catenin regulation. Besides Jade1, SIAH1 induces ubiquitination of non-phosphorylated β-catenin too in response to the tumor suppressor p53 to promote β-catenin degradation [41].

In the Wnt-on state, β-catenin translocates into the nucleus due to the disassembly of the β-catenin destruction complex. Recently, researchers found that β-catenin ubiquitination in the nucleus are mainly mediated by c-Cbl and tripartite motif-containing protein 33 (TRIM33). C-Cbl is a Ring finger ubiquitin E3 ligase expressed in endothelial cells, which binds to the nuclear active non-phosphorylated β-catenin via the armadillo-repeat region. Indeed, c-Cbl also mediates unbiquitination of cytoplasmic β-catenin in a similar way to Jade1 [42]. TRIM33 is another E3 ubiquitin ligase targeting nuclear active β-catenin and acts regardless of Wnt signaling activation [43]. Phosphorylation of β-catenin at Ser715 by protein kinase Cδ (PKCδ) initiates TRIM33-mediated destabilization.

Tribbles homolog 2 (TRIB2), a member of Tribbles protein family, is found to be a direct target gene of Wnt/β-catenin and promotes HCC cell survival and transformation. Interestingly, a negative feedback loop exists between TRIB2 and Wnt signaling whereby TRIB2 facilitates the ubiquitination of TCF4 and β-catenin [44]. β-TrCP, COP1 and Smurf1 are the TRIB2-associated E3 ligases. The four proteins scaffold into a complex with each component acting as an essential and indispensable inhibitor of Wnt activity. However, the direct relationships between the three E3 ligases and β-catenin have not yet been investigated. The ubiquitination of β-catenin is not always promoting its degradation. The E3 ubiquitin ligase identified by differential display (EDD) was discovered in recent years, which leads to an enhanced stability of β-catenin via ubiquitinating β-catenin through Lys29- or Lys11-linked ubiquitin chains [45]. Moreover, this process requires the presence of GSK3β, which until then had always been shown to be a negative modulator in the Wnt/β-catenin pathway.

Just like kinases and phosphatases oppose each other in the regulation of phosphorylation, there are also deubiquitinases that oppose ubiquitin ligases in regulating β-catenin ubiquitination. Ubiquitin-specific peptidase 7 (USP7) mediates deubiquitination of β-catenin and enhances canonical Wnt signaling [46]. Through siRNA library screening, Ubiquitin-specific peptidase 47 (USP47) is identified to activate Wnt signaling by deubiquitinating β-catenin in the C-terminus and inhibiting its degradation [47]. Recently, ubiquitin-specific protease 4 (USP4) was identified as a deubiquitinating enzyme for β-catenin, which supports the stability of β-catenin by deubiquitinating the phosphorylated β-catenin and retards the nuclear translocation of β-catenin [48].

Unlike phosphorylated sites Ser33, Ser37 and Thr41, phosphorylation of some sites in β-catenin helps maintaining its stability. For instance, phosphorylation of β-catenin on Ser675 by PKA enhances β-catenin stability through inhibiting its ubiquitination [49, 50]. Moreover, Tyr654 phosphorylation of β-catenin by RTK reduces its interaction with E-cadherin, facilitates phosphorylation at Ser675 and increases Wnt signaling. These studies suggest that RTK and PKA inhibitors serve as efficient therapeutic strategies for patients with intestinal or other cancers with active Wnt/β-catenin signaling.

### The interplay of autophagy and β-catenin signaling

Autophagy is an evolutionarily highly conserved catabolic pathway that degrades cellular macromolecules and organelles. Recent studies indicate that there exists a crosstalk between Wnt/β-catenin signaling and autophagy. On one side, the Wnt/β-catenin signaling pathway acts as a negative regulator of autophagy. Under nutrient rich conditions, autophagy is maintained at basal level only. β-Catenin limits autophagosome formation and functions as a transcriptional co-repressor of p62 through binding to the transcription factor TCF4 [16]. In multiple myeloma cells, β-catenin is also reported to inhibit autophagy. β-catenin silencing increases the expression of LC3 and Beclin 1 as well as the activation of AMPK, a major activator of autophagy [51]. A recent study indicates that glucose deprivation triggers PKC-dependent β-catenin proteasomal degradation, which induces autophagy [52]. On the other side, β-catenin itself is targeted for autophagic clearance in autolysosomes upon autophagy induction. Under nutrient deprivation conditions, β-catenin is selectively degraded via forming a complex with LC3, attenuating β-catenin/TCF4-driven autophagy inhibition so as to favor adaptation during metabolic stress [16]. Wang et al reported that activation of PKCι, an oncoprotein in esophageal squamous cell carcinomas, enhances β-catenin accumulation by suppressing autophagy. Meanwhile, activation of autophagy promotes autophagic degradation of β-catenin [53]. In addition, Wnt signaling is negatively regulated by autophagy through degradation of other Wnt signaling proteins. Dishevelled proteins transduce canonical Wnt signals to the GSK3β-destruction complex, resulting in the stabilization of β-catenin. Starvation induces localization of Dvl2 to autophagosomes for degradation so as to negatively regulate Wnt signaling. The reverse correlation between Dvl expression and autophagy is observed in late stages of colon cancer, indicating the malfunction of autophagy may contribute to the aberrant activation of Wnt signaling in tumor formation [54]. These studies suggest that the positively or negatively reciprocal regulation between β-catenin stability and autophagy play a critical role in tumorigenesis controlled by Wnt/β-catenin signaling.

## REGULATION OF β-CATENIN SUBCELLULAR LOCALIZATION

Nuclear concentration of β-catenin is the hallmark of Wnt/β-catenin signaling. As a consequence of aberrant activation of Wnt signaling, the active form of β-catenin translocates into the nucleus to act as a transcriptional activator. There exist specific regulatory mechanisms governing the subcellular translocation of β-catenin, independent of its stability.

The nuclear envelope is a double membrane permeated by nuclear pores. Proteins larger than 50 kDa with the nuclear localization sequence (NLS) or the nuclear export sequence (NES) require active transport by transport receptors (including importins and exportins) via the nuclear pore complexes (NPC), which are composed of 30-50 different nucleoporin proteins. Fagotto et al. demonstrated that although β-catenin does not contain NLS, it still could be imported into the nucleus by directly binding to the nuclear pore machinery. The characteristics of β-catenin are similar to importin-β, whose nuclear transport does not require additional components of the transport machinery, such as importins, exportins or RAN-GTPase [55]. This functional similarity might due to the structural similarity of β-catenin and importin-β, which share 8–13 related 42-amino-acid armadillo repeats that permits a direct interaction with the nuclear pore proteins. However, another group challenges this model. They found no evidence of a direct interaction between β-catenin and nucleoporins [56]. Therefore, whether there is an interaction between β-catenin and nucleoporins in the context of the tumor environment still needs further clarification.

**Figure 3:**
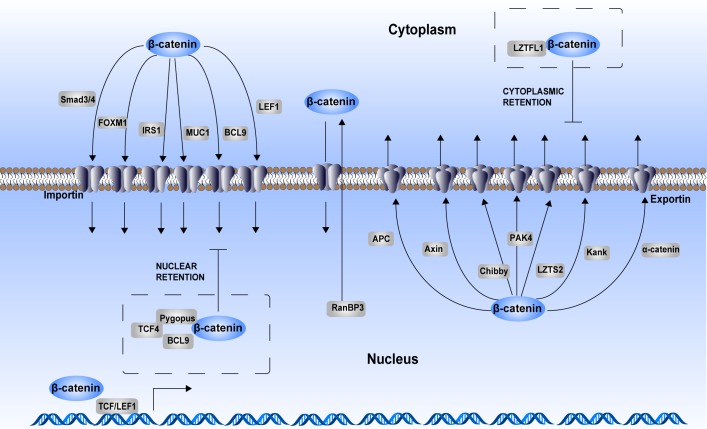
Regulation of β-catenin cytoplasmic–nuclear trafficking As a consequence of aberrant activation of Wnt signaling, β-catenin translocates into the nucleus to act as a transcriptional activator. Although β-catenin itself does not have a nuclear localization sequence (NLS), its armadillo repeats mediate its nuclear translocation process. Many chaperones participate in the regulation of β-catenin cytoplasmic–nuclear trafficking. Smad3/4, FOXM1, IRS1, MUC1, BCL9 and LEF1 are chaperones that assist nuclear translocation of β-catenin in an importin-dependent manner. APC, AXIN, Chibby, PAK4, LZTS2, Kank, and α-catenin are chaperones that assist with β-catenin cytoplasmic translocation in an exportin-dependent manner. RanBP3 also enhances β-catenin nuclear export, while this process is independently of exportin. In addition, some proteins form complex with β-catenin to maintain its cytoplasmic or nuclear retention. LZTFL1 binds with β-catenin to sequester it in the cytoplasm. TCF4–Pygopus–BCL9 complex helps enhancing β-catenin nuclear retention.

Except for direct interaction with NPC, many nuclear import or export chaperones mediate the subcellular translocation and nuclear retention of β-catenin [57] (Figure 3). Smad3/4, FOXM1, MUC1, IRS1, BCL-9 and LEF1 act as the import chaperones. The export chaperones include Exportin1-dependent ones, such as APC, AXIN, Chibby, PAK4, LZTS2, Kank and α-catenin, and Exportin1-independent ones, such as RanBP3. Moreover, the TCF4–Pygopus–BCL9 complex helps enhancing the β-catenin nuclear retention.

In addition to the chaperones that directly interact with β-catenin and assist in its translocation, some oncoproteins or tumor suppressors regulate the shuttling process in an indirect way. N-myc downstream-regulated gene 1 (NDRG1), a potent metastasis suppressor, inhibits EMT in prostate and colon cancer cells. Overexpression of NDRG1 in colon cancer cells inhibits the nuclear localization of PAK4, increasing the levels of membrane-associated β-catenin and downregulating β-catenin transcriptional activity [58]. Custos is another protein reported to be a component of canonical Wnt signaling [59]. Custos binds to β-catenin in a Wnt responsive manner without affecting its stability, and the interaction is required to promote β-catenin nuclear translocation during organizer formation. Although the mechanism has not been elucidated, one possibility is that Custos modulates the accessibility of β-catenin to the nuclear pore complex. Leucine zipper transcription factor-like 1 (LZTFL1) has been identified as a novel tumor suppressor in gastric cancer [60]. It inhibits the EMT of gastric cancer cells by inhibiting β-catenin nuclear translocation without affecting its protein expression. However, the precise mechanism still needs to be determined.

In general, post-translational modification of β-catenin itself or in coordination with the import or export chaperones directly regulate the subcellular localization of β-catenin. In addition, many upstream regulators of those chaperones also influence the nuclear translocation of β-catenin. Besides, some proteins promote β-catenin retention in the nucleus or cytoplasm through a direct protein–protein interaction manner. Most of the regulators play critical roles in tumorigenesis and tumor development, providing new targets for cancer treatment.

## REGULATION OF THE TRANSCRIPTIONAL ACTIVITY OF β-CATENIN

As a protein with no DNA binding ability, β-catenin is thought to form a transcriptional complex with TCF/LEF, p300/CBP, and other proteins. It functions as a transcriptional activator following nuclear translocation. Studies show that the complex is diverse and complicated in different types of cancers. More and more co-activators and regulators have been found, which involve and influence Wnt/β-catenin transcriptional activity.

### The transcription complex of β-catenin

TCF proteins have DNA-binding domains, but no intrinsic transcriptional activity. In the absence of β-catenin, TCF4 interacts with Groucho/TLE, CtBP and histone deacetylase proteins, which leads to promoter repression. Once monoubiquitylated by X-linked inhibitor of apoptosis (XIAP), Gro/TLE dissociates from TCF/Lef, thereby allowing β-catenin-TCF/Lef complex assembly and promoting Wnt-specific transcriptional program [61]. In the β-catenin-TCF4 dimer, the C-terminus of β-catenin provides the transactivation domain, whereas TCF4 provides the DNA-binding domain, thereby forming a transcriptional switch. Besides interacting with TCF/LEF, β-catenin also binds to p300/CBP, TATA-box binding protein (TBP), Pontin52, Reptin52, Brg-1, MUC1-C, SOX10, p68/p72, FOXM1, yes-associated protein 1 (YAP1) and FBW1, which are all involved in tumor development or progression.

Homologues p300 and CBP are histone acetyltransferases (HATs), which recruit basal transcriptional machinery to the promoters. The C-terminus of β-catenin interacts directly with the CH3 domain of p300, thus alleviating the repression of Wnt-target genes induced by Groucho/TLE and so on [62]. Furthermore, p300 induces acetylation of β-catenin at Lys345, which enhances β-catenin-TCF4 binding affinity. Acetylation of Lys345 specifically increases the transcriptional activity of β-catenin in a TCF-dependent manner [63]. SWI/SNF and Rsc are two highly related, but distinct, ATP-dependent chromatin-remodeling complexes. The two complexes use Brg-1 as a common component, which has ATPase activity. Through interaction with Brg-1, β-catenin recruits SWI/SCF or Rsc-like complexes to TCF target gene promoters, facilitating the chromatin remodeling that is a prerequisite for efficient transcriptional activation [64]. However, in apparent contradiction to this, Brg-1 is reported to be a tumor suppressor in both breast and colon cancer cells, and suppresses *CyclinD1* gene expression [65, 66]. The mechanisms behind these contradictory phenomena are still unknown. Pontin52 is reported with a high expression in colon cancer and promotes COX-2 expression. Indeed, Pontin52 binds with TBP and β-catenin to enhance the β-catenin transcriptional activity [70]. While, the homologous interacting partner Reptin52/TIP49 also interacts with TBP and β-catenin, but plays an opposite role. Two segment polarity genes in Drosophila, *legless* (the homolog of human BCL9) and *pygopus* are positive regulators of the Wnt/β-catenin signaling pathway [68]. BCL9 acts as an adaptor linking pygopus to the nuclear β-catenin-TCF complex. Pygopus and legless are essential transcriptional coactivators of β-catenin [69]. The leukemia-associated protein, Mllt10/Af10-Dot1, is identified as another component of the β-catenin-TCF transcriptional complex in colorectal cancer to promote transcription elongation [70]. p68 and p72 are homologous proteins in the DEAD box family of RNA helicases that are overexpressed in colon cancer. p68 and p72 form complexes with β-catenin to activate β-catenin target gene transcription [71]. Except for augmenting β-catenin transcriptional activity, cytoplasmic p68 and p72 protect β-catenin from degradation by dissociating β-catenin from the APC-Axin-GSK3β complex. Moreover, depletion of p68 and p72 in colon cancer cells results in decreases in cell proliferation and expression of target genes such as *c-Myc* and *CyclinD 1*. SOX10 is a member of the SOX protein family and its expression is upregulated in HCC. Interestingly, SOX10 expression is positively correlated with elevated β-catenin levels in HCC, and β-catenin activity promotes oncogenic effects of SOX10. SOX10, TCF4 and β-catenin form a ternary complex, which binds to similar DNA sequences as TCF4 via a single HMG domain, and facilitates the binding of TCF4 with β-catenin [72]. β-TrCP1/Fbw1 is a component of the Skp1-Cullin-Rbx1/F-box E3 ubiquitin ligase complex, which facilitates β-catenin degradation. However, β-TrCP1/Fbw1 enhances the transcriptional activity of β-catenin through binding with p300 and β-catenin, and co-localizing with the promoters of β-catenin target genes in colon cancer [73]. Hence, β-catenin recruits additional transcription factors to modulate its transcriptional activity in a protein–protein interaction (PPI) manner (Table 1).

**Table 1:** Transcriptional cofactors of β-catenin

Transcriptional cofactors	Negative or positive regulators	Binding site in β-catenin	Mechanisms
**TCF family** [61]	Positive	Arm3-8 repeats	Binds to promoters of target genes.
**P300/CBP** [62]	Positive	C-terminus	Acetylates histone H3 to promote transcription; acetylates β-catenin to enhance the interaction between β-catenin and TCF4.
**Brg-1** [64]	Positive	Unknown	Facilitating chromatin remodeling as a prerequisite for efficient transcriptional activation.
**Pontin52/CBP** [67]	Positive	Unknown	Binds with TBP and β-catenin to promote transcription.
**Reptin52/TIP49** [67]	Negative	Unknown	Plays opposite roles compared to Pontin52/TBP.
**BCL9** [68]	Positive	Arm 1 repeat	Acts as an adaptor linking Pygopus to the nuclear β-catenin-TCF complex to promote transcription.
**Mllt/Af10-Dot1** [70]	Positive	C-terminus	Essential for transcription elongation.
**p68/p72** [71]	Positive	Arm 3-8 repeats	Binds to promoters of target genes.
**SOX10** [72]	Positive	Unknown	Forms a ternary complex with TCF4 and β-catenin.
**βTrCp1/Fbw1a** [73]	Positive	Interacts with P300	Interacts with p300 and β-catenin, and co-localizes to the promoters of β-catenin targets.
**FOXOM1** [123]	Positive	Arm 11-12 repeats	Forms a ternary complex with TCF4 and β-catenin.
**Yes-associated protein 1** [7]	Positive	Unknown	Forms a ternary complex with TBX5 and β-catenin.

**Table 2:** Therapeutics targeting the Wnt/β-catenin signaling pathway

Agents	Classification	Targets
**Targeting of Wnt ligands or receptors**		
**LGK974 I** [124]	Small molecule	Inhibiting Lipid modification of Wnt by targeting PORCN.
**OMP-54F28** [103]	Antibody	An inhibitor of Wnt ligands.
**OMP-18R5** [103]	Antibody	An inhibitor of Wnt receptor-multiple frizzleds.
**OTSA101** [103]	Antibody	An inhibitor of Wnt receptor-frizzled-10.
**Targeting of β-catenin stability**		
**Celecoxib** [110]	Small molecule	Celecoxib dampens the auto-phosphorylation of the tyrosine kinase receptor c-Met, which results in the activation of GSK3β and degradation of β-catenin.
**DIF1/3** [112]	Natural product	Activating GSK3β by reducing its Ser9 phosphorylation to degrade β-catenin.
**Genistein** [111]	Natural product	Activating GSK3β to degrade β-catenin.
**G007-LK** [105]	Small molecule	Stabilizing Axin-2 by inhibiting TNK1/2 to promote degradation of β-catenin.
**XAV939** [104]	Small molecule	Stabilizing Axin-2 by inhibiting TNK1/2 to promote degradation of β-catenin.
**JW55** [106]	Small molecule	Stabilizing Axin-2 by inhibiting TNK1/2 to promote degradation of β-catenin.
**Pyrvinium** [107]	Small molecule	Activating CK1 to promote β-catenin degradation.
**Targeting of β-catenin nuclear translocation**		
**WGA** [113]	Small molecule	Suppressing nuclear transport of β-catenin by inhibiting NPC.
**Targeting of β-catenin transcriptional activity**		
**PRI 724** [120]	Small molecule	Specifically downregulating the expression of β-catenin-TCF-responsive genes by interrupting β-catenin/CBP.
**PKF115-584** [115]	Small molecule	Interrupting β-catenin/TCF to inhibit target genes’ transcription.
**CGP9049090** [115]	Small molecule	Interrupting β-catenin/TCF to inhibit target genes’ transcription.
**Vitamin D** [118]	Natural product	Competing with TCF and LEF for β-catenin binding.
**Retinoid acid** [119]	Natural product	Competing with TCF and LEF for β-catenin binding.
**SAH-BCL-9** [114]	Peptide	Interrupting β-catenin/BCL-9 to inhibit target genes’ transcription.

### Regulators of the β-catenin transcriptional complex

The co-activators above either have chromatin remodeling capacities, transcriptional activities or have direct DNA-binding abilities. Besides, they all interact with β-catenin or other components to form a complex and enhance its transcriptional activity. In addition, some regulators directly bind to β-catenin, interrupting or enhancing its interaction with other components of the complex. Tumor adaptation to hypoxia is mainly mediated by two transcription factors, the hypoxia-inducible factor 1α or 2α (HIF1α or HIF2α). HIF1α competes with TCF4 for direct binding to β-catenin at the promoter region of HIF1α target genes. β-Catenin enhances HIF1α-mediated transcription, thereby promoting cell-cycle arrest, cell survival and adaptation to hypoxia [74]. Like HIF1α, HIF2α interacts with β-catenin, but at a different site. HIF2α is found to assemble with β-catenin and TCF to facilitate β-catenin target gene transcription and enhance cell proliferation [75]. The opposite actions of HIF proteins suggest that the HIF1α/HIF2α balance may determine cell fate when hypoxia and Wnt stimulation coexist. Besides, the microenvironment is critical to determine the transcriptional regulation.

Many proteins interact with components of the transcriptional complex to regulate β-catenin transcriptional activity. Here, we focus on the regulators of TCF4 and p300/CBP. The TCF4-related regulators include HOXB13, SOX4, RUNX3, CDK8, TCTP and Daxx. HOXB13 is exclusively overexpressed in the normal prostate and the colorectum tissues. HOXB13 is a negative regulator of TCF4 that promotes TCF4 degradation. A lower HOXB13 expression in colorectal cancer confers TCF4-mediated transactivation [76]. On the contrary, SOX4 activates β-catenin/TCF4 transcription by upregulating TCF4 at the transcriptional level without a direct β-catenin association [77]. As a tumor suppressor, RUNX3 inactivation occurs in many cancer types, especially in more than 80% of gastric cancers. RUNX3 forms a ternary complex together with TCF4 and β-catenin, attenuating the DNA-binding activity of β-catenin/TCF4, independent of its own DNA-binding activity [78]. Cyclin-dependent kinase 8 (CDK8) is an oncogene that couples transcriptional regulators to the basal transcriptional machinery, and is implicated in the transcriptional regulation of key pathways involved in colon cancers. CDK8 promotes binding of β-catenin/TCF to the promoter of β-catenin targets implicated in cancer, including *MYC* [79]. Transcription factor E2F1 is a negative regulator of β-catenin/TCF activity. CDK8 and E2F1 both present at the MYC promoter, CDK8 suppresses the E2F1-dependent inhibition of β-catenin-mediated transcription [80]. Together, these observations suggest that therapeutic interventions targeting CDK8 may confer a clinical benefit in β-catenin-driven malignancies. Translational-controlled tumor protein (TCTP), a multifunctional protein, promotes cell growth, tumor progression, and inhibits apoptosis. TCTP directly interacts with TCF4 and enhances the binding affinity of β-catenin to TCF4, leading to the expression of Wnt target genes and glioma cell proliferation [81]. Daxx acts as a transcriptional co-regulator in multiple cell signaling pathways, and was reported to interact with several transcriptional factors, such as hormone receptors, p53, Smad4 and NF-κB. Daxx associates with TCF4 in DNA-bound complexes and co-upregulates the β-catenin/ TCF4-mediated gene expression in colon cancer cells [82]. The molecular basis of this process might contribute to the weakened interaction between TCF4 and Groucho/TLE or to the recruitment of CBP to the β-catenin/TCF4 complex. The concrete molecular mechanisms remain to be elucidated.

p300/CBP-related regulators include HIF2α, KLF4 and Smad2. As mentioned above, HIF2α activates β-catenin by recruiting p300 through its transactivation domains. KLF4 is a transcription factor highly expressed in the adult intestine. In contrast to HIF2α, KLF4 was demonstrated to directly interact with the C-terminal transactivation domain of β-catenin and block p300/CBP recruitment by β-catenin. KLF4 inhibits p300/CBP-mediated β-catenin acetylation as well as histone acetylation on Wnt target genes, resulting in the inhibition of Wnt signaling [83]. SMAD2, a pivotal downstream effector of TGF-β signaling, interacts with TCF4, β-catenin and p300 [84]. It functions as a co-activator of canonical Wnt/β-catenin signaling, independent of Smad4, through the histone acetyltransferase activity of p300. This function occurs in the presence of the β-catenin/TCF4 complex, demonstrating a mechanism how Smad2 functions as a tumor promoter in the late stages of colon cancer.

## TARGETING WNT/β-CATENIN SIGNALING AS AN ANTI-CANCER STRATEGY

### Wnt/β-catenin signaling in cancer

Associations between Wnt/β-catenin signaling and cancer initiation, tumor growth, tumor metastasis, tumor dormancy, tumor immunity and tumor stem cell maintenance have been revealed over the last two decades. Constitutive activation of Wnt/β-catenin signaling induced by mutations of genes, or aberrant activation of Wnt receptors trigger tumorigenesis in the colon, liver, skin, breast, bone marrow, and maybe more tissues [85, 86]. It is indicated that Wnt/β-catenin signaling maintains populations of tumor-initiating cells or cancer stem cells [87, 88]. Mechanistically, Wnt/β-catenin signaling pathway upregulates the expression of cell cycle-related proteins, such as Cyclin B1 and Cyclin C to promote carcinogenesis and development of HCC [89]. When talking about tumor growth, Wnt/β-catenin signaling may play opposing roles in different tumor tissues. It has been reported that inhibition of β-catenin signaling could suppress pancreatic tumor growth by disrupting the nuclear β-catenin/TCF1 complex [90]. In addition, β-catenin is sufficient to enhance the growth of myeloma cells in vitro and of prostate tumors in mouse models [91, 92]. However, Wnt-3a and β-catenin inhibit the growth of human and mouse melanoma cells grafted in mice, which gives a new cellular activity to the Wnt/β-catenin pathway [93]. Wnt/β-catenin also drives cancer metastasis in tumors such as breast cancer, HCC and melanoma [9, 94, 95]. Although Wnt/β-catenin signaling promotes tumor metastatic spread by increasing migration and/or invasion in most cases, it inhibits migration and brings about a lower proliferation index while inducing metastasis in the case melanoma, adding to the complexity of this topic [93, 96]. Tumor dormancy is a major reason for tumor relapse, often occurring many years after the removal of a primary tumor. β-Catenin has been shown to upregulate urokinase plasminogen activator (uPA) expression in colorectal tumors to promote invasion, metastasis and dormancy in human colorectal tumors [97]. In a study about latency competent cancer (LCC) cells, they found that autocrine DKK1 secretion induced attenuated Wnt signaling is associated with LCC quiescence [98].

Wnt/β-catenin signaling also participates in the regulation of tumor immunology. By analyzing 266 metastatic human cutaneous melanoma samples, Spranger et al. revealed that tumor-intrinsic β-catenin activation dominantly excludes T cell infiltration into the melanoma tumor microenvironment. Therefore, intrinsic β-catenin activation may represent one mechanism of primary resistance to T cell-based immune-oncology therapies [99]. Cross-priming helps to generate anti-tumor CD8^+^T cells. β-Catenin expression in DCs negatively regulates antitumor immunity by inhibiting their cross-priming ability resulting in a dampened CD8^+^ T cell response [100]. Hence, the Wnt/β-catenin signaling pathway may be a potential target for anti-tumor immunotherapy.

Accumulating evidences suggest that Wnt/β-catenin signaling helps maintaining the stemness of cells in the embryo and the adult, as well as in cancer. The mammalian intestine contains a crypt compartment maintained by multipotent stem cells and a villus compartment extending into the lumen. Wnt signaling controls the proliferation of stem cells within the crypt base and thus drives the emergence of cells through the transit-amplifying zone into the villus. Indeed, Wnt target genes, such as LGR5, CD44 and Msi1, have become well-established markers of intestinal stem cells [11]. In the pathogenesis of colorectal cancer, Wnt/β-catenin signaling promotes the tumorigenesis in both the “top-down” model, in which differentiated cells repossess pluripotency and produce aberrant crypt foci that later turn into adenomas, and the “bottom-up” model, in which the stem cells at the crypt base hyperproliferate, migrate upwards and act as tumor-initiating cells [88]. Using the mouse mammary tumor virus (MMTV)-induced breast cancer model, scientists found that Wnt signaling may induce the expansion of stem-like cells during mammary tumor progression [101, 102]. As Wnt/β-catenin signaling greatly promotes the differentiation of many cancer stem cells, which can be precursors of mature cancer cells, it is of great significance to develop therapeutic strategies targeting this pathway to reduce the stem cell behavior. Although a number of small molecules inhibiting this pathway are being developed, some of which have been in preclinical or early clinical study, whether these molecules can reduce stem cell ratio and/or activity in patients awaits confirmation.

### Therapeutics against cancers by targeting Wnt/β-catenin signaling

**Figure 4:**
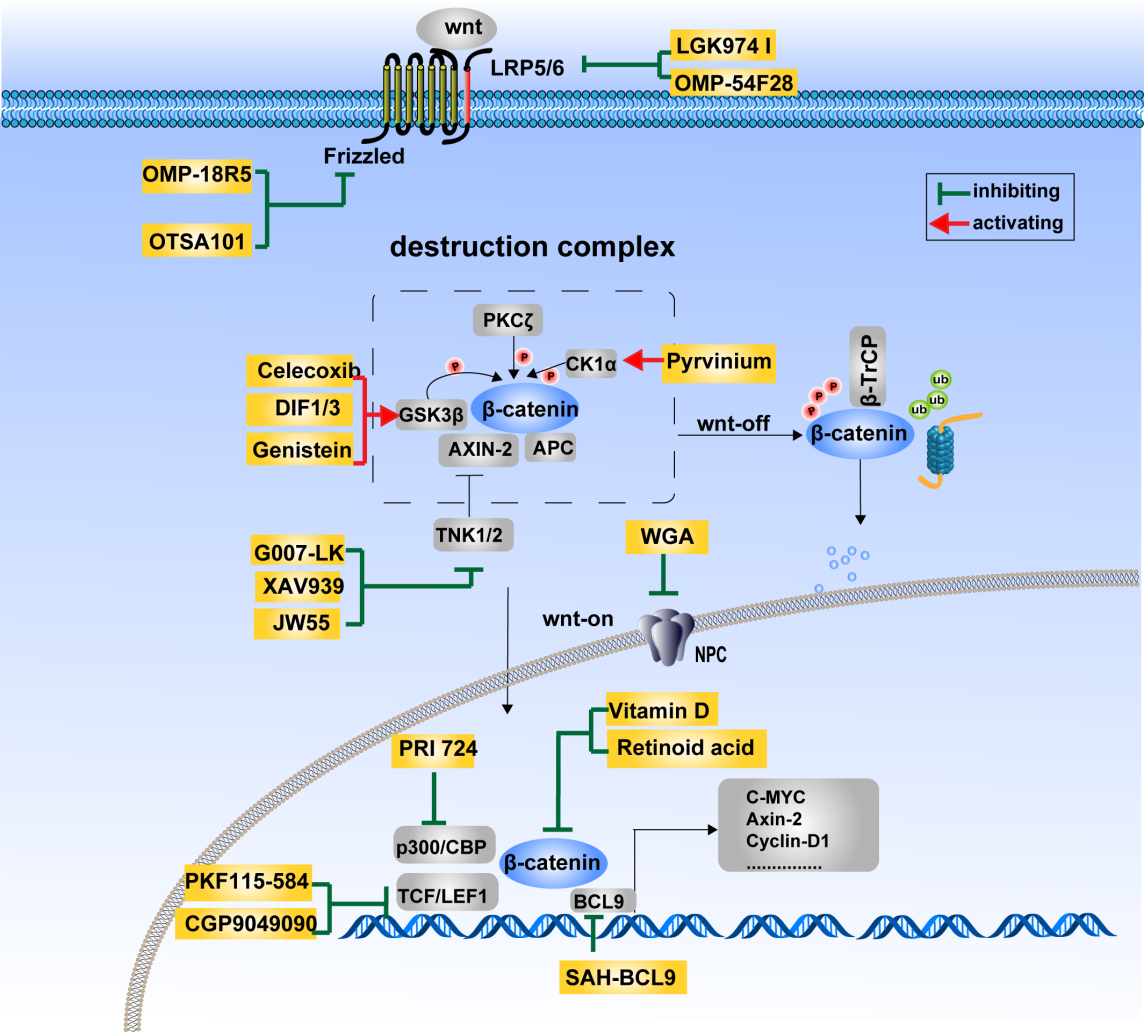
Therapeutics targeting the Wnt/β-catenin signaling pathway The Wnt/β-catenin signaling pathway is at the center of carcinogenesis and tumor progression. Therapeutics targeting this pathway is of great potential in the development of anti-cancer drugs. The agents currently being investigated mainly targets Wnt ligands and receptors (LGK974 I, OMP-54F28, OMP-18R5, OTSA101), the β-catenin destruction complex (Celecoxib, DIF1/3, Genistein, Pyrvinium, G007-LK, XAV939, JW55), β-catenin cytoplasmic–nuclear translocation (WGA) and β-catenin transcriptional complexes (PRI 724, Vitamin D, Retinoid acid, PKF115-584, CGP9049090, SAH-BCL9).

The Wnt/β-catenin pathway has been proved to be crucial to various kinds of carcinoma. Therapeutic strategies targeting this signaling pathway are increasingly being proposed to aid the fight against cancer. Although no drugs specifically inhibiting this signaling pathway have been approved for the market, considerable efforts have been made towards their development. Inhibitors of the Wnt/β-catenin pathway can be classified into five categories according to the properties of the drugs: small molecules, peptides, antibodies, RNA interference and natural compounds. Figure 4 illustrates the potential therapeutic strategies employed against cancer, based primarily on the three regulatory mechanisms of β-catenin activity discussed above.

#### Wnt ligand and receptor modulators

In the Wnt/β-catenin signaling pathway, one target is the ligand—Wnt itself, which controls the activity of the pathway. Lipid modification of Wnt by membrane-bound o-acyltransferases-porcupine (PORCN) promotes its secretion and its binding ability with cognate receptors. LGK974, a potent and specific small-molecule inhibitor of PORCN demonstrates efficacy at well-tolerated doses in multiple tumor models, such as murine breast cancer and human head and neck squamous cell carcinoma in vivo. The phase I clinical study of LGK974 in patients with malignancies dependent on Wnt ligands is ongoing. In opposition to the role of Wnt-3a, Wnt-5a promotes the degradation of β-catenin. The formylated 6-amino-acid peptide fragment, (Foxy-5), mimics the effects of Wnt-5a and is currently being tested in a phase I clinical trial. Antibodies targeting the Wnt ligands and receptors are another method by which this signaling pathway is inhibited. OMP-18R5, OTSA-101 and OMP-54F28 are antibodies against multiple frizzleds, frizzled-10 and Wnt ligand respectively, and have all been enrolled in Phase I clinical trials [103].

#### The modulators of β-catenin degradation

Axin, GSK3β and CK1 participate in the regulation of β-catenin degradation. Efforts are made to activate kinase activity and thus to reduce the stability of β-catenin. Axin is an important protein in promoting β-catenin degradation. Tankyrase1/2 is a kinase destabilizing Axin, which is chosen as a target to reduce the accumulation of β-catenin. G007-LK and JW55, inhibitors of TNK1/2, cause stabilization of Axin2 and thus increase degradation of β-catenin. The two small molecules are now in preclinical research. XAV939, another Axin stabilizer, has been in the phase I clinical trial to treat subjects with CRC, unfortunately it was stopped due to low enrollment [104–106]. As pivotal kinases destabilizing β-catenin, GSK3β and CK1 are also chosen as drug targets. Pyrvinium is a small molecule that activates CK1 to promote β-catenin degradation [107]. Recent studies show that non-steroidal anti-inflammatory drugs (NSAIDs) prevent the development of polyps and reduce tumor size in familial adenomatous polyposis patients through different mechanisms [108]. Firstly, NSAIDs reduce the expression of Cox-2, which is highly expressed in various cancers. Secondly, Cox-2-derived PGE2 activates the Wnt-signaling cascade at the level of β-catenin degradation through cAMP/PKA-mediated phosphorylation to increase stemness and progenitor populations [109]. Thirdly, drugs like celecoxib dampen the auto- phosphorylation of the tyrosine kinase receptor c-Met, which results in the activation of GSK3β and degradation of β-catenin [110]. Lastly, differentiation-inducing factor (DIF) and Genistein, a soy-derived isoflavone and phytoestrogen, have also been reported to promote β-catenin degradation by activating GSK3β [111, 112].

#### The modulators of β-catenin subcellular localization

Therapeutics targeting β-catenin cellular distribution is a promising potential strategy for the inhibition of Wnt/β-catenin pathway, although few inhibitors have yet been identified. One strategy would be to disturb the function of NPC, as β-catenin is transported through NPC during its subcellular localization either via a direct interaction or via an active, receptor-mediated mechanism. Wheat germ agglutinin (WGA), an inhibitor of NPC, has been reported to suppress nuclear transport of β-catenin [113]. However, the NPC is the conduit for numerous proteins translocating to the nucleus, so universal inhibitors targeting the NPC have a fundamental lack of specificity, which might interrupt normal cell homeostasis. Despite this, searching for small molecules specifically disturbing the interaction of β-catenin and NPC proteins is a potential future method.

In addition to NPC, nuclear import chaperones of β-catenin, such as Smad3/4, FOXM1, MUC1, IRS1, BCL-9 and LEF1, are also potential targets. Small molecules, especially peptides designed to interrupt the PPI of β-catenin and its specific import chaperones in specific tumor types, reduce the accumulation of β-catenin in the nucleus and suppress its transcriptional activity. For example, a peptide targeting BCL-9, an import chaperone as well as a transcriptional co-activator of β-catenin, has been developed [114] (discussed later). Besides molecules targeting the β-catenin nuclear transportation process, therapeutics enhancing the cytoplasmic retention or disturbing the nuclear retention of β-catenin might also be good potential choices.

#### The modulators of the β-catenin transcriptional complex

High frequency of Wnt pathway gene mutations enhances the β-catenin transcriptional activity independent of upstream factors, which would limit the applicability of some therapeutic modalities mentioned above. Therefore, more efforts have been made to directly attenuate the function of β-catenin in the nucleus. As β-catenin functions as a transcriptional activator in a complex with TCF4, P300/CBP, BCL9 and so on, small molecules targeting these PPIs have a potentially bright future. More than thirty compounds and a stapled α-helical peptide have been reported to interfere with the β-catenin–TCF4 interaction [115–117]. The crystal structure analysis revealed that β-catenin utilizes the same groove of a large surface (3700 Å^2^) to bind with TCF4, Cadherin and APC. Moreover, the dissociation constant (Kd) of the β-catenin–TCF PPI is much lower than those of the β-catenin-cadherin and β-catenin–APC PPIs, making it difficult to obtain a highly selective inhibitor. β-Catenin function loss in cadherin junctions increases cell migration and induces metastasis. Furthermore, dissociation of APC from β-catenin results in its nuclear accumulation. These facts emphasize that obtaining high selectivity is a major concern for drug development. Recently, J. Leon Catrow et al have discovered a series of compounds with much higher selectivity for the β-catenin–TCF interaction over β-catenin–Cadherin and β-catenin–APC interactions. For example, PKF115-584 and CGP049090, which are obtained through optimization of the Acyl Hydrazone Moiety [115]. This is a good starting point from which to discover more and more high-value selective molecules to disrupt the β-catenin–TCF PPI. In addition, Vitamin D and retinoid acid have been shown to compete with TCF and LEF for β-catenin binding [118, 119]. Other than TCF, β-catenin also recruits transcriptional coactivators such as cyclic AMP response element-binding protein (CBP) or its closely-related homolog, p300. Katayoon H. Emami et al have discovered a selectively low molecular-weight inhibitor (ICG-001 or PRI 724) targeting β-catenin-CBP, but not β-catenin-p300, which specifically downregulates the expression of β-catenin-TCF-responsive genes [120]. Phase I and II clinical trials of ICG-001 for acute and chronic myeloid leukemia were completed in October 2015. This provides a new perspective on the selection of PPI targets. BCL9 is another good target, which helps Wnt to upregulate the stemness of cells in the colon epithelium and in adenocarcinomas [121]. Takada et al developed a stabilized α helix of BCL9 (SAH-BCL9), disassembles native β-catenin-BCL9 complexes and selectively suppresses Wnt transcription, with anti-tumor effects consistent with this mechanism [114]. Thus targeting the β-catenin-TCF interaction is not the only means to inhibit the transcriptional activity of β-catenin, indicating that more fundamental mechanistic studies need to be undertaken in order to deepen our knowledge in this field. Given the gene mutations of Wnt pathway components present in numerous malignancies, drug therapies aimed at disrupting the β-catenin transcriptional complex seem to be a rational, effective and potentially universal strategy against cancer.

## CONCLUSIONS AND FUTURE PERSPECTIVES

Based on early discoveries linking the activation of Wnt/β-catenin signaling to HCC, breast and colon carcinomas, it has generally been accepted that aberrant activation of Wnt signaling promotes tumor initiation and progression. The activity of β-catenin is under the control of several highly regulated processes, including ligand–receptor interactions, β-catenin stability, β-catenin nuclear translocation and β-catenin transcriptional activity. Future studies aimed at determining the mechanisms that control these processes will be necessary to identify pivotal signaling nodes that could be targeted by therapeutic interventions.

The complexity of the Wnt signaling pathway offers multiple levels of therapeutic intervention and currently there are a plethora of interventional approaches being explored to reduce Wnt/β-catenin signaling output. For example, great efforts have been made to interrupt upstream Wnt signaling events, including Wnt lipidation, Wnt secretion, and ligand-receptor interactions [122]. Some compounds have already reached clinical trials to assess their applicability as cancer therapeutics. However, sometimes such inhibitors targeting upstream signaling events may prove ineffective due to downstream mutations. Hence, targeting the downstream effectors of the Wnt/β-catenin signaling pathway in the nucleus might be more effective. For example, efforts have been taken to inhibit the nuclear transport of β-catenin, especially by interrupting its interaction with the nucleoporin proteins. Although no candidate compound has been brought out at the present stage due to the issue of specificity, we believe that targeting the nuclear transport of β-catenin holds therapeutic potential and deserves further investigation. Furthermore, small molecules and stapled peptides designed to disrupt LEF-1–β-catenin, CBP–β-catenin or BCL9–β-catenin transcription factor complexes can disrupt the final effector state of β-catenin and exhibit great promise for treating cancers.
